# Quantitative evaluation of precautions against the COVID-19 indoor transmission through human coughing

**DOI:** 10.1038/s41598-022-26837-0

**Published:** 2022-12-30

**Authors:** Zhenguo Nie, Yunzhi Chen, Meifeng Deng

**Affiliations:** 1 Department of Mechanical Engineering, Tsinghua University, Beijing, 100084 China; 2 State Key Laboratory of Tribology in Advanced Equipment, Beijing, 100084 China; 3 Beijing Key Lab of Precision/Ultra-precision Manufacturing Equipments and Control, Beijing, 100084 China; 4 Marine Engineering College, Dalian Maritime University, Dalian, 116026 Liaoning China; 5 State Key Laboratory of Vegetation and Environmental Change, Institute of Botany, Chinese Academy of Sciences, Beijing, 100093 China; 6 University of Chinese Academy of Sciences, Beijing, 100049 China

**Keywords:** Computational models, Biomedical engineering, Disease prevention

## Abstract

In this work, we focus on the dispersion of COVID-19-laden droplets using the transient computational fluid dynamics (CFD) modeling and simulation of the coughing process of virus carriers in an enclosure room, aiming to set up the basic prototype of popular precautionary strategies, i.e., face mask, upward ventilation, protective screen, or any combination thereof, against the indoor transmission of COVID-19 and other highly contagious diseases in the future. A multi-component Eulerian–Lagrangian CFD particle-tracking model with user-defined functions is utilized under 8 cases to examine the characteristics of droplet dispersion concerning the mass and heat transfer, droplet evaporation, air buoyancy, air convection, air-droplet friction, and turbulent dispersion. The result shows that implementing upward ventilation is the most effective measure, followed by wearing face masks. Protective screens can restrict the movement of the coughing droplets (though it will not reduce viral load). However, applying protective screens arranged with lean can be counterproductive in preventing the spread of COVID-19 when it is inappropriately placed with ventilation. The soundest solution is the combination of the face mask and upward ventilation, which can reduce the indoor infectious concentration by nearly 99.95% compared with the baseline without any precautionary strategies. With the resumption of school and work in the post-epidemic era, this study would provide intelligence-enhancing advice for the masses and rule-makers to curb the pandemic.

## Introduction

The novel coronavirus disease 2019 (COVID-19) has spread rapidly around the world, sending billions of people into lockdown. To date (August 12, 2022), COVID-19 has had over 584 million cases and 6.41 million deaths reported to World Health Organization (WHO). The closure of educational institutions and workplaces in an attempt to contain the spread of the COVID-19 pandemic is impacting millions of students and employees. This pandemic has made clear the fundamental role of airborne droplets and aerosols as potential viral carriers in the indoor environment. From this, many promising strategies have come into service against the infectious transmission route, both in short and long-range^1^. Face masks, head-mounted shields, and protective screens are three common strategies for cutting off droplet transmission in the short term, whose limitation mainly is air leakage due to material defects and misguided usage^2^. For the long-distance precautions, although air ventilation and air purification are multi-functional ways of improving air quality and controlling contamination concentration, their efficacy would be tempered because of the manifold decorations and house types. It is advisable to use some simultaneously to overcome these limitations of precautions. However, there needs to be more theoretical guidance and a quantitative description of the choice of the combined strategies in indoor scenarios. Under such circumstances, when social activities are necessary, systematic precautions are urgently demanded to build multi-layers of protection in indoor public.

**Figure 1:**
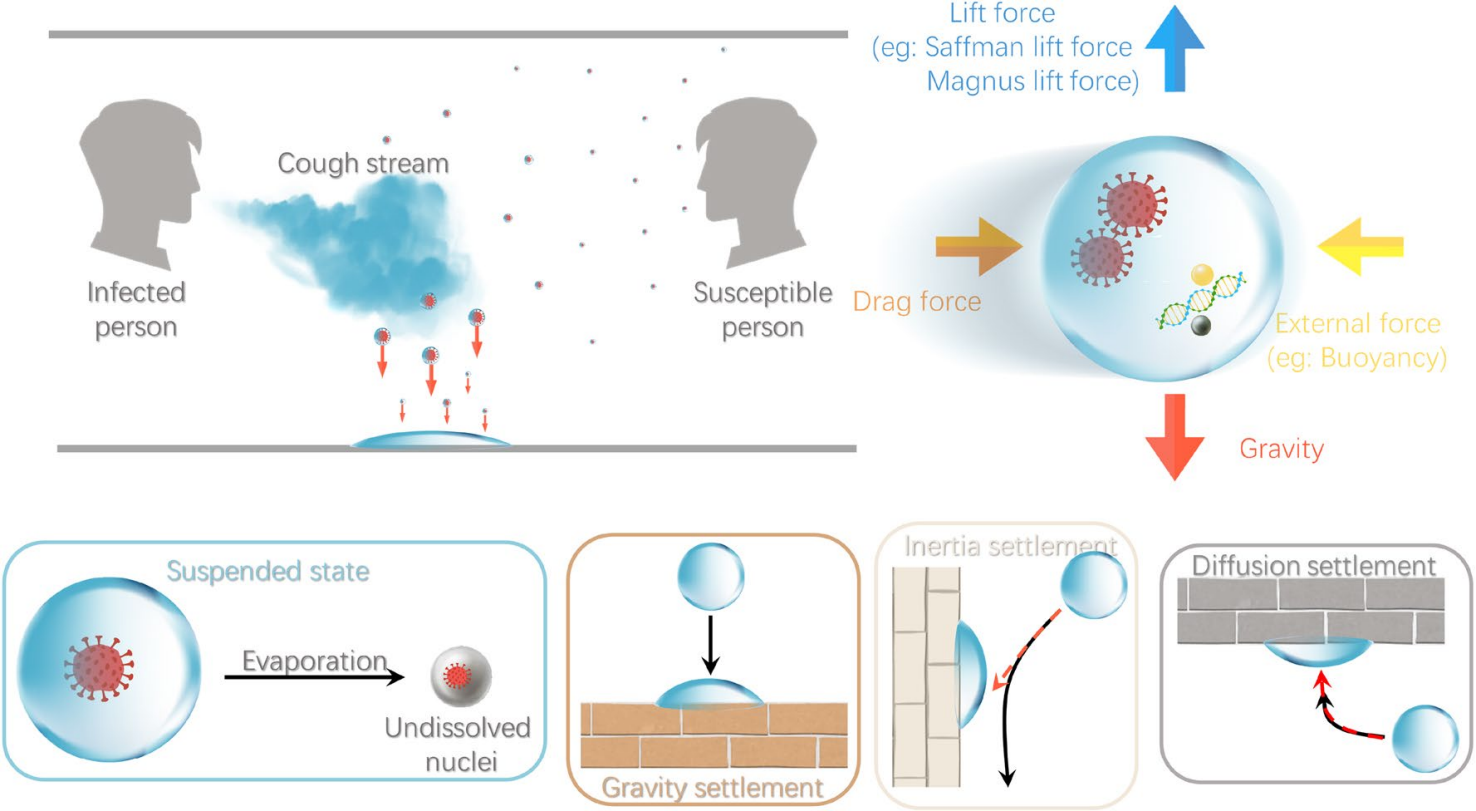
Diffusion and settlement of cough droplets cloud. The figure was drawn in Adobe Illustrator (version 16.0) and Microsoft PowerPoint (version 16).

The current research has mainly conducted the efficacy of each strategy separately. For short-term precautions, Kahler^3^ declared all types of masks can reduce the front through-flow of exhaled flow, which reduced the harm in face-to-face circumstances; Ho^4^ quantified the risks of exposure under face mask and face shield; both Dbouk^5^, and Pendar^6^ analyzed the transmission of droplets through a face mask filter with detail. For long-distance precautions, Yang^7^ made an elaborate comparison of the efficacy of the advanced air distribution methods in removing airborne contaminants; Dai^8^ estimated the association between infection probability and ventilation rate based on the Wells–Riley model, whose accuracy needs further discussion^2^; Alsaad^9^ showed personalized ventilation could penetrate the corona-shaped thermal boundary layer to encase the human body, providing clean air for inhalation; Zhang^10^ conducted a parametric study of air change per hour (ACH) rate, and concluded that the stronger air movement would generally prevent direct aerosol inhalation. However, none of these studies adopted a combination of more infectious control strategies. Most of them applied a less comprehensive fluid physics model, which led to the deviation or omission of the natural coughing and ambient characteristics, e.g., non-evaporable materials in saliva^5,11^, human body heat^6^, coughing initial direction^9^, droplet size distribution^8^, and transient mass flow rate of droplet cloud^4^. All the strategies mentioned above have proven to directly change the airborne transmission of a cough cloud. However, the fluid dynamic mechanisms of indirect pathogen transfer between people through exhaled aerosol remain poorly understood^12–14^. Li et al.^15^ applied a combination of the VOF model and the DPM model to simulate the fluid flow and the movement of particles. Redrow et al.^16^ provided insight into how the composition of the sputum droplet directly affects its evaporation and condensation during transmission. Much research conducted on droplet evaporation^17^, speed^18^, and trajectory^11^ have been limited to the scope of a simplified physical model, as the motion mechanism of the single-particle. As shown in Fig. 1, the evolution of each droplet is mainly under the governance of gravity force, buoyancy, air resistance, and external forces. The large droplets are expected to move like projectiles before settlement. In contrast, the smaller droplets can bring the virus into the airflow and hence cause infectious diseases, and finally suspended in the air or diffused to walls rather than falling onto the ground. However, due to significant variance in the data among different subjects and trial conditions, the basic flow pattern of the droplet swarm is affected and is hard to determine^19^.

Although the experimental study is the most intuitively reliable way to explore the coughing flow pattern, it is still difficult to capture the whole process of a coughing event in quantization, which releases multi-phase turbulent flows that are generally composed of buoyant hot moist air and suspended droplets of various sizes^20^. The characteristics of expiratory airflow (e.g., maximum reach distance and maximum velocity) can be influenced by individual physiological differences such as age, gender, and vital capacity^21^. Bourouiba^22^ indicated that any indoor violent expiration events (i.e., coughing, sneezing, talking) could spread droplets extending up to 7 m–8 m. Lee^23^ adopted a particle sizer and an optical particle spectrometer to measure the cough particle concentration of 10 patients with cold symptoms in real time. Results showed that transmission could spread more than 3 m. Zhu^24^ ensured that saliva is expelled at speeds up to 22 m/s during each coughing by using a particle image velocity (PIV) system, while Han^25^ utilized PIV to conclude 15.2/13.1 m/s of peak velocity of male/female. To sum up, even though much experimental research has been devoted to investigating human coughing, their data on coughing characteristics still need to be consistent with each other, which brings barriers to fixing initial conditions and verification criteria on CFD studies.

CFD has significant potential for helping health services against the COVID-19 pandemic indoor transmission during social activities. Compared to the experimental study, CFD is more widely used due to its lower cost and higher efficiency, as well as it can provide a deeper insight^26,27^ with more detailed outputs. To quantify the coughing process with and without infection prevention and control, researchers adopted the “droplet lifetime” and “propagation range”^28,29^ as reference designators in guiding the recommended social distancing. Centers for Disease Control and Prevention (CDC) claimed that people should stay 6 feet ($\sim \hbox {1.8}$ m) away from others^30^, which is much insufficient based on recent research. Rosti^31^ followed the position and evaporation of the coughing airflow by massive state-of-the-art numerical simulations. They found that airborne-transmitted droplets can travel less than 2.5 m versus more than 7.5 m horizontally. Muthusamy^32^ concluded that a significant fraction of unhindered coughing spreads beyond the 2 m. Even though when wearing masks with intake/exhaust air streams and other ventilation system features, there may still result in a small percentage ($<0.5\%$) of particles reaching distances cross the threshold of 2 m. Considering that droplets contain involatile components, organics, and inorganics, the infectious droplet nuclei can suspend for a long time by the entrainment of surrounding flow. Issakhov^33^ noted that the indoor social distance of 2 m is performed for simple breathing but not enough under coughing or sneezing scenarios. Van^34^ experimentally investigated how long the COVID-19 virus remains viable within saliva or aerosols, which indicated that the lifetime of the survival droplets is 1.1–1.2 h during the 3-h whole observation time. During such a long time, infectious nuclei could spread over the entire confined space^35^. On this occasion, appropriate social distance is still being determined when people stay indoors long enough with poor air exchange.

In this work, we present a modified Eulerian–Lagrangian droplet spray model with sufficient coughing characteristics, aiming to describe the airborne transmission of cough saliva particles from an infector sitting in a closed room with and without various precautions. Experimental results validate the proposed model well. By embedding the evaporation model, we can evaluate the infectious concentration by analyzing the Lagrangian-discrete phase remaining over distance and time via accurate CFD simulation. The properties of coughing (implanted by user-defined commands) and precautions are based on the widely-used or generally-agreed data. The initial droplet size follows the Rosin–Rammler distribution. The coughing angle, direction, transient mass flow rate, non-evaporable mass fraction, and mask filtration efficiency are from up-to-date experimental studies. Upward ventilation is adopted as one of the advanced total volume air distribution (TVAD). The protective screen is simplified based on the commonly-used style in school libraries or canteens. In short, the modeling of the transient coughing process is much more complete than those in the current literature.

Our main contributions are:

1. With and without popular precautionary strategies (i.e., face mask, upward ventilation, protective screen, or any combination thereof), we calculate the propagation distance and distribution characteristics of cough droplets to compare the efficacy of the precautionary strategies. Precautious guidance of infectious control layouts and social distance are given for closed indoor scenarios.
2. A generalized multi-component Discrete Phase Model based on the Eulerian–Lagrangian approach with user-defined commands is proposed and employed to realize a mechanism modeling of coughing puffs. To our knowledge, the proposed model is equipped with the enhanced transient feature of coughing progress and is more comprehensive in characterizing the evaporation and dispersion of airborne droplets and aerosols for a coughing event.

## Modeling method and numerical simulation

As an enclosed chamber flow problem, three-dimensional, time-dependent, non-isothermal, and multi-phase Navier–Stokes equations, in conjunction with the Reynolds-averaged Navier–Stokes (RANS) turbulence model, are applied to model the transmission of the aerosol particle in the indoors, taking a normal classroom as the example in this study. The air is the Eulerian phase under the control of the continuous govern equations, while the saliva is the Lagrangian phase, the discrete govern equations. The volume-of-fluid (VOF) method is employed to capture the fluid–fluid interface, of which dynamics are explicitly described. Running the gamut of droplet evaporation, air buoyancy, air convection, air-droplet friction, turbulent dispersion, Brownian motion of nuclei, and settlement on the surface, our study aims to set up the basic prototype of popular precautions by quantitatively describing the transmission of the virus-laden droplets of human coughing. ANSYS Fluent 2019 R2^36^ is used as the CFD solver with the mesh generator from ANSYS Workbench.

Eight infectious control schemes, including three single strategies and their combinations, are simulated for a duration of 60 s. Considering there are many people who wear the N95 masks improperly or wear the less prophylactic masks in real life, the gauze mask is selected here to be close to life rather than the ideal situation of wearing an N95 mask as much current research. As one of the advanced TVADs, upward ventilation is commonly used but lacks studies compared to the others. The protective screen after the revised design possessed an oblique angle vertical to the coughing jet, aiming to induce the saliva to move upward and away from the range of human activity. The results show that the soundest strategy is the combination of the face mask and upward ventilation.

### Geometry and meshing

**Figure 2:**
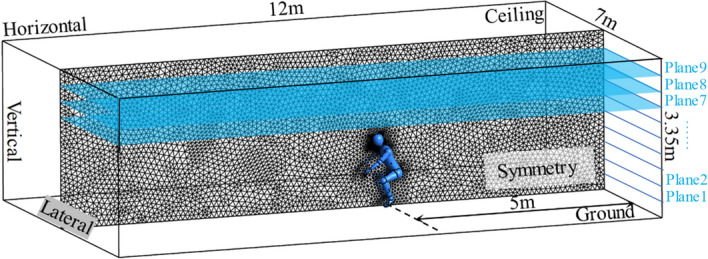
The computational domain dimensions and vertical planes. The figure was drawn in Tecplot 360 (version 2016 R2) and Adobe Illustrator (version 16.0).

**Figure 3:**
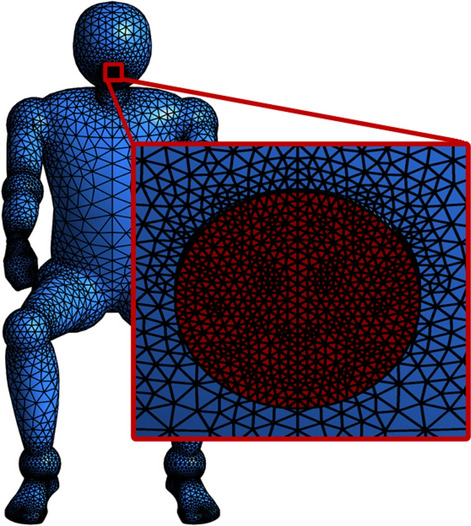
Computational mesh with refinement near the mouth. The figure was drawn in Tecplot 360 (version 2016 R2) and Adobe Illustrator (version 16.0).

This study represents a typical situation of airborne respiratory disease transmission: a COVID-19 infector is coughing inside a regular-sized classroom with the dimension of $7 \times 3.35 \times 12$ m$^{3}$, which can also represent an analogous closed public indoor workspace. Default names of the main boundaries with computational mesh are displayed in Fig. 2. The infected person is sitting in the lateral center of the classroom, as well as 5 ${\textrm{m}}$ away from the back surrounding wall. The domain is halved by setting a horizontal symmetry plane to save computational costs. The ambient temperature is 20 $^{\circ }$C, while its relative humidity is $62\%$, which are the average values of the authorized winter climate record of Washington D.C.^37^. The coughing process of the infector is modelled as a cone-atomized spray loaded with virus-laden droplets, which are released from the opening mouth 2 ${\textrm{cm}}$ in diameter^6^. The computational domain is discretized by unstructured tetrahedron grids with an average skewness number of 0.2 and an average surface y+ near 3. Twelves inflation layers with a growth ratio of 1.1 are generated near all wall boundaries to capture detailed velocity and pressure gradient change at the near wall regions. The refinement applied in the vicinity of the mouth is shown in Fig. 3. The number of droplet trajectories was also analyzed. Consistent with the findings of Dudalski^38^ and Busco^39^, the angle between the coughing direction and the horizontal plane is $\theta = 27.5^{\circ }$, and the spreading angle of the coughing cone spray is $\alpha = 12.5^{\circ }$ (shown in Fig. 6). The simulated time duration is 60 $\textrm{s}$ with a non-uniform time step for the continuous phase of $\Delta t = 0.01$–0.2 s. The time step of the discrete phase is $\Delta t = 5 \times 10^{-3}\,{\textrm{s}} \sim 0.01 \,{\textrm{s}}$. The 3D Reynolds-averaged Navier–Stokes (RANS) equations are solved with the boundary conditions and RNG k-e turbulence model through ANSYS Fluent.

**Figure 4:**
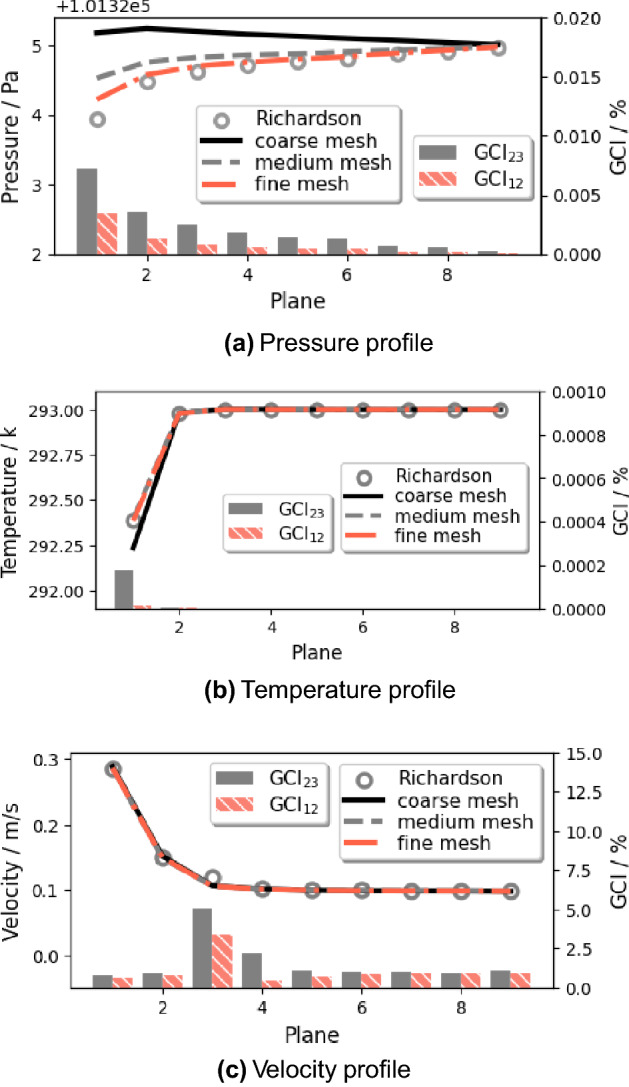
Grid independence study for three uniformly refined grids.

**Figure 5:**
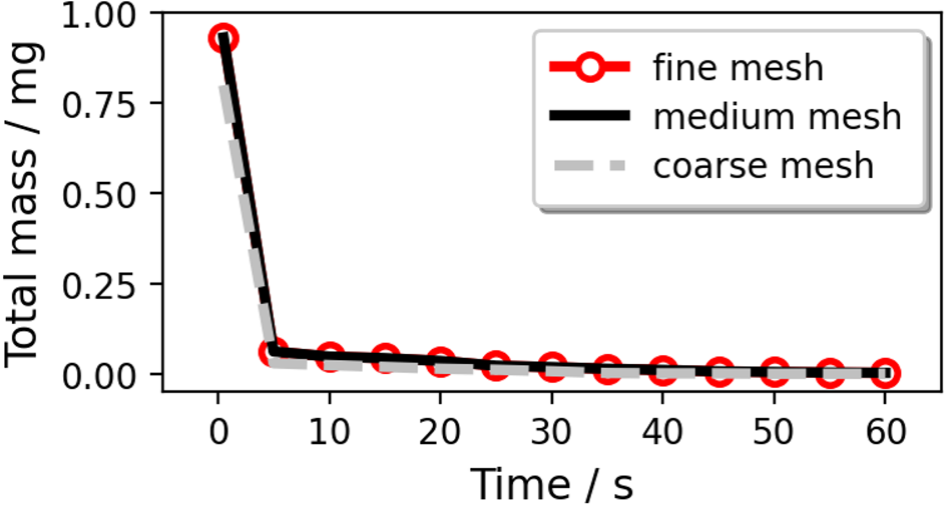
Variation in the total mass of coughing droplets varying the mesh number.

The Grid Convergence Index (GCI)^40^ applied with the Richardson Extrapolation is employed in the grid independence study for the Eulerian phase to estimate the refinement error caused by distinct grid spacing. The space discretization of the domain in half is performed using coarse, medium, and fine meshes of 75, 212, and 597 thousand elements with a linear refinement factor of $\sqrt{2}$. Figure 4 shows the profiles of the mass-flow averaged pressure, temperature, and velocity at nine cross-section planes (shown in Fig. 2) along the height direction. The values calculated from Richardson extrapolation are the theoretical values of an infinitely fine grid scheme with zero spacing. The GCI is calculated with a safety factor of 1.25 between two grids: $\hbox {GCI}_{12}$ considers the change from the fine grid to the medium grid and $\hbox {GCI}_{23}$ from the medium grid to the coarse grid. The results show a limited dependence of the mass flow-averaged pressure, velocity, and temperature with the maximum local $\hbox {GCI}_{23}$ value of 0.0034%, 0.03%, and 0.0001% at Plane 3 (in Fig. 4a), Plane 1 (in Fig. 4b), and Plane 1 (in Fig. 4c), respectively. For the validation of the Lagrangian model, the total mass of cough droplets over time is calculated to check the mesh independence (in Fig. 5). The results of the medium and fine mesh almost overlap, while the predicted value of the coarse mesh is low. It is concluded that the fine mesh is sufficient to extract quantitative data for both the Eulerian and the Lagrangian phases from the simulation.

In Fig. 7, the baseline (Case 1) is conducted as the situation where droplets from a regular cough are spreading in an enclosed area. Various precautions, namely, face mask (Case 2), air-applied (Case 3), and protective screen(Case 4), are placed separately to alter the original pattern of cough, further reducing the infection risk. The rest of the cases (Case 5–8) are different combinations thereof, as shown in Table 1.

**Figure 6:**
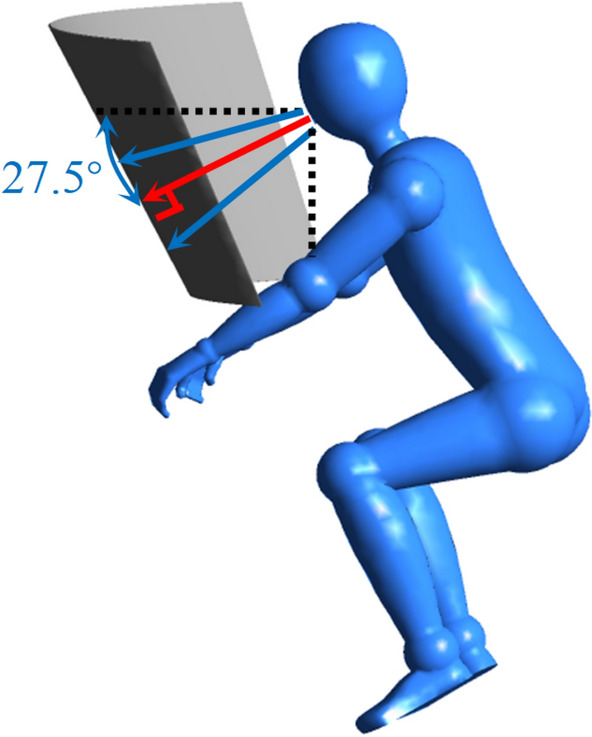
Schematic of the spreading angle of the cough particles. The figure was drawn in Tecplot 360 (version 2016 R2) and Adobe Illustrator (version 16.0).

Discrete Phase Model (DPM)^41^ integrated with the evaporation model^42^ is adopted to calculate the transmission and evaporation process of discrete droplet particles under the continuous air ambient. As shown in Table 2, two sets of boundary conditions are considered: the general cases with a quiescent interior ambient can allow droplets to diffuse freely (in Case 1/2/4/7), and the ventilated cases with fresh air transported from upward ventilation can discharge the virus-laden aerosol (in Case 3/5/6/8). The definitions of boundaries in DPM are trap, escape, and reflect. For the “trap” setting, the trajectory calculations are terminated, and the droplets will settle on the wall; under the “reflect” condition, the droplets will rebound off the wall; when droplets “escape” from the wall, their trajectory calculations are stopped, and their entire mass will be removed from the computational domain. The static parts (i.e., screen, surrounding wall) are set to be wall boundaries, which are treated as no-slip in CFD selection. While in DPM selection, once the droplets contact these walls, the trap condition is triggered, and the DPM trace of the droplet particles aborts. To address human plumes, previous studies^43,44^found that when neglecting the radiation and latent heat, the convective heat load of a human being is about 36W, which is equivalent to a heat flux of 22.83 W/m$^{2}$ set on the surface of the human body. Referring to the survey^45^, approximately 85% of the measurements of the averaged wind speeds in the indoor workplace with ventilation are below 0.3 m/s. For the ventilated cases, the classroom ground is transferred to a velocity inlet (0.1 ${\mathrm{m/s}}$), and the classroom ceiling is a pressure outlet (101,325 ${\textrm{Pa}}$). At the same time, droplets under the DPM trace will trap at the inlet and escape at the outlet. Temperature and humidity are assumed to be the same as the background setting (293 K and 62%).

**Table 1:** Cases of the pandemic precautions investigated in the present work.

Case No.	Face mask	Upward ventilation	Protective screen
1	No	No	No
2	Yes	No	No
3	No	Yes	No
4	No	No	Yes
5	Yes	Yes	No
6	No	Yes	Yes
7	Yes	No	Yes
8	Yes	Yes	Yes

**Figure 7:**
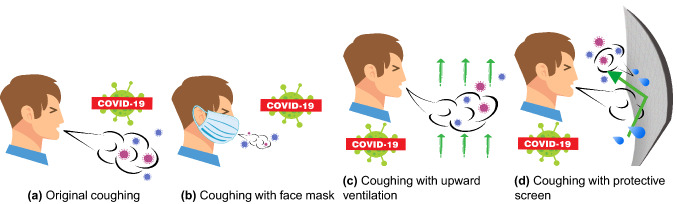
Anti-epidemic measures. The figures were drawn in Adobe Illustrator (version 16.0).

**Table 2:** CFD and DPM selections for computation.

Boundary	General cases	Ventilated cases
Surrounding wall	**CFD: Wall**	**CFD: Wall**
	temperature = 293 K	temperature = 293 K
	**DPM: Trap**	**DPM: Trap**
Human body	**CFD: Wall**	**CFD: Wall**
	Heat flux = 22.83 $\mathrm W/m^{3}$	Heat flux = 22.83 $\mathrm{W/m}^{3}$
	**DPM: Trap**	**DPM: Trap**
Classroom ground	**CFD: Wall**	**CFD: Velocity inlet**
	temperature = 293 K	temperature = 293 K
	**DPM: Trap**	velocity = 0.1 m/s
		humidity = $62\%$
		**DPM: Trap**
Classroom ceiling	**CFD: Wall**	**CFD: Pressure outlet**
	temperature = 293 K	temperature = 293 K
	**DPM: Trap**	pressure = 101325 Pa
		humidity = 62$\%$
		**DPM: Escape**
Protective screen (if exists)	**CFD: Wall**	**CFD: Wall**
	temperature = 293 K	temperature = 293 K
	**DPM: Reflect**	**DPM: Reflect**

**Table 3:** Initial constituents of complete sputum droplets.

References and this paper	Percentage of water (%)	Non-volatile species
Redrow et al.^16^	93.5$\%$	Protein, Lipid, Carbo-hydrate, DNA, Salt
Yan et al.^46^	$\sim$95	Salts, Glycoproteins, Lipids
Nicas et al.^47^, Yan et al.^48^, Li et al.^49^	$\sim$98.2	$Na^{+}$, $K^{+}$, $Cl^{-}$, Lactate, Glycoproteins
This paper	$\sim$98.2	Salt, Protein, Carbo-hydrate, Lipid, DNA, COVID-19 virus

### Coughing features and simulation strategy

**Figure 8:**
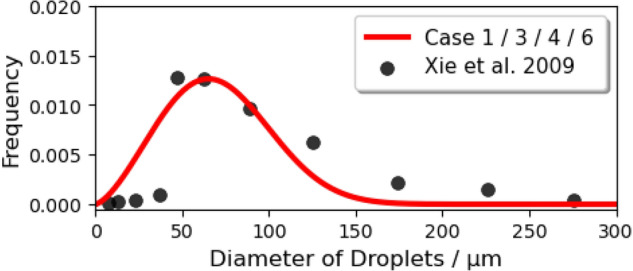
Initial saliva droplets size distribution (ranging from 1 to 300 micrometer).

**Figure 9:**
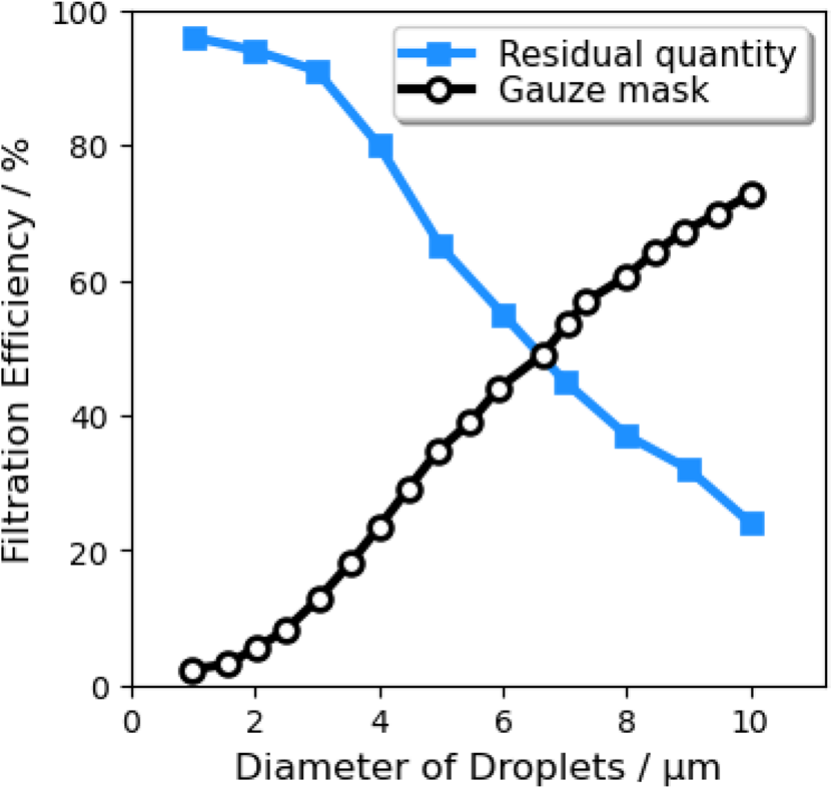
Gauze mask filtration efficiency and residual droplet quantity.

**Figure 10:**
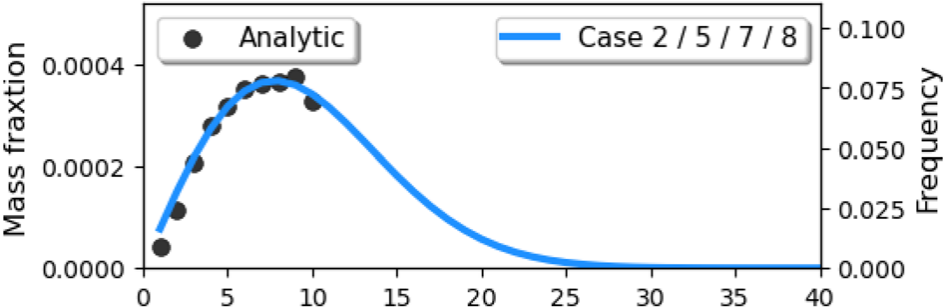
Sectional saliva droplets size distribution (intercepting from 1 to 10 micrometer).

**Figure 11:**
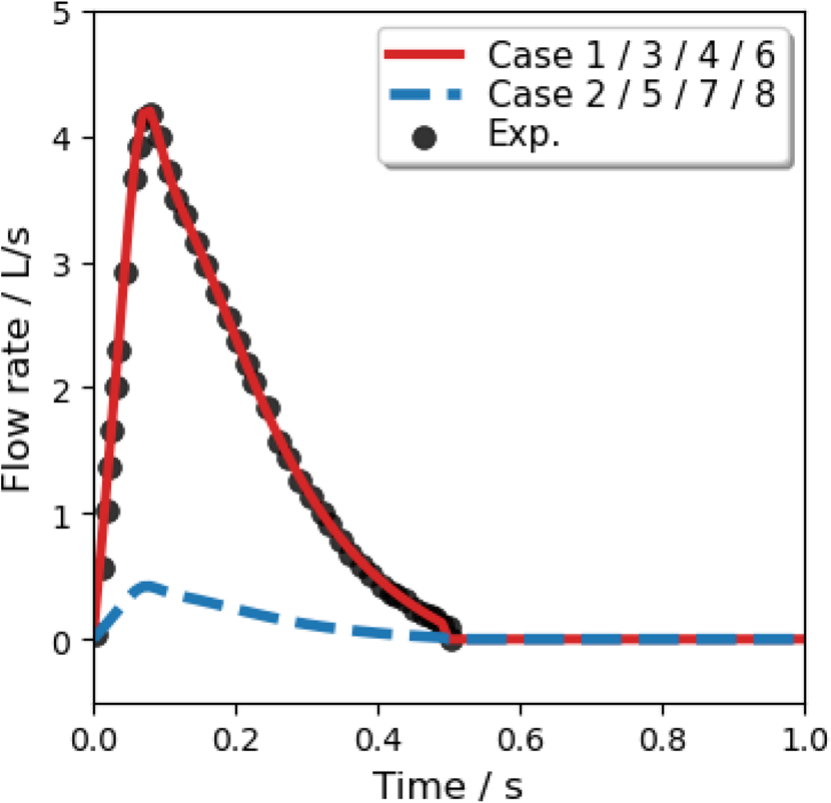
Mass flow rate of a single cough.

We assumed that: the nuclei of saliva are insoluble and would not crystallize during the evaporation process; droplets remain spherical under drag; the temperature of a single droplet is uniform. For the normal cough in Case 1/3/4/6, many experiments were conducted to measure the size distribution of droplets^50–52^. The experimental data from Xie et al.^53^) was corrected near the origin of the injection, which is reliable^5^ and is fitted by a Rosin–Rammler distribution in the range of 1–300 micrometer (Fig. 8). It was found that when the trajectory number was larger than 20,000, the predicted vapor concentration field was free from the number^49^. In order to investigate the scenario of wearing a face mask, a gauze filtering face-piece respirator is mimicked to be positioned above the mouth of the manikin in Case 2/5/7/8 by employing the realistic filtration efficiency (as shown in Fig. 9^54^). Therefore, the diameter distribution of droplets in a normal cough duration is reconstructed with values ranging from 1 to 40 $\upmu$m (Fig. 10). The time-dependent mass-flow rate of a cough behavior within a duration of 0.5 s (discrete by Yan et al.^48^, based on the experimentally measured data in^55^) with Rosin–Rammler distribution is implanted by user-defined functions and is fitted as the red line in Fig. 11. In addition, the blue dashed line represents the face mask situation and is approximately $10\%$ the value in the normal group.

**Figure 12:**
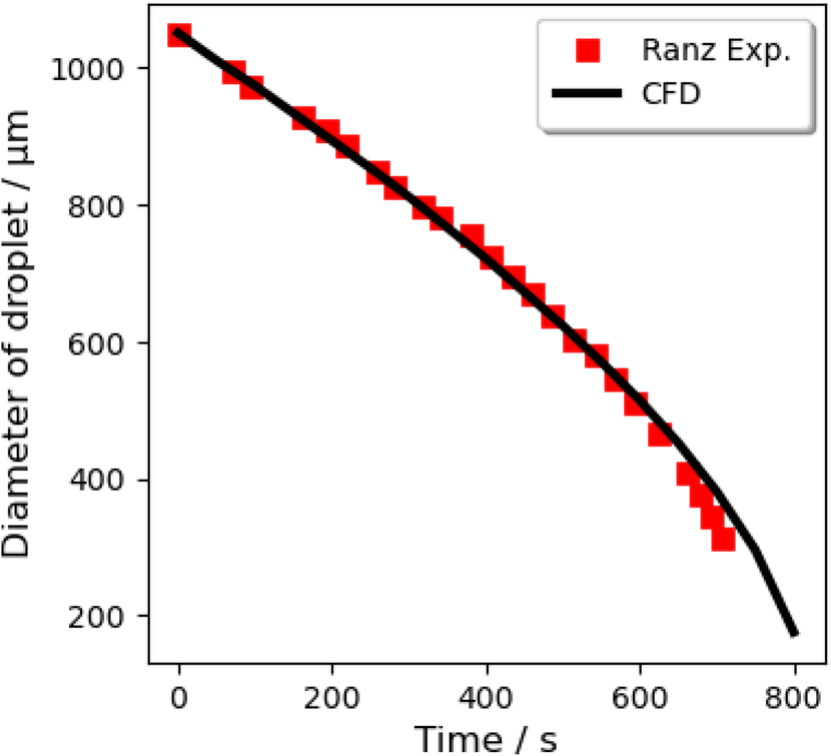
Experimental validation of CFD model by saliva droplets size change^56^.

**Figure 13:**
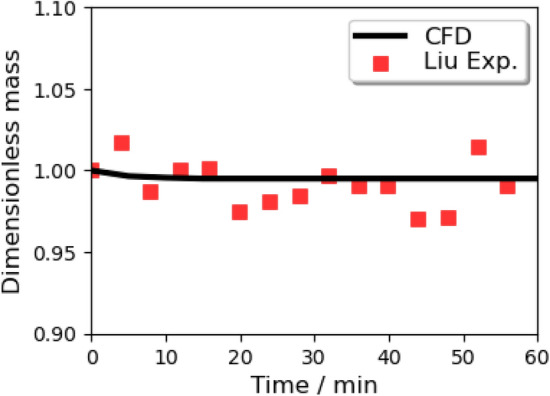
Dimensionless mass losses of water droplets at RH = 96%^57^.

**Figure 14:**
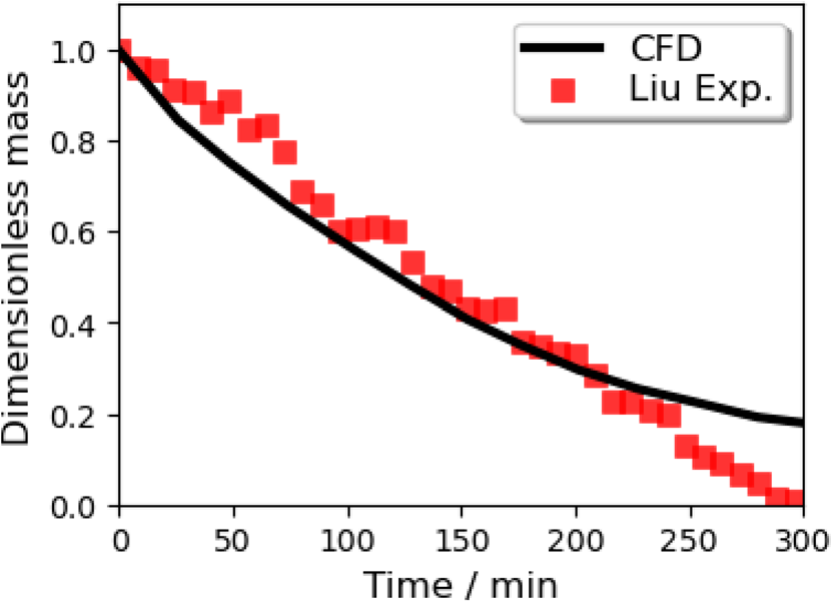
Dimensionless mass losses of water droplets at RH = 84%^57^.

### Physical models

#### Governing equations of continuous phase

For the CFD model^36^, multi-phase flow with gas and liquid is modelled by an Eulerian–Lagrangian frame. For simulating the humidity, the ambient air is an Eulerian n continuous gas phase and is selected as a multi-component of dry air and water vapor. As a homogeneous mixture, the thermal dynamic properties $\phi _{mix}$ ($C_p, \rho , \mu , \lambda , etc.$) of the gas phase are calculated by the mass fraction of the mixture species components^39^:

1 $$\begin{aligned}{} & {} \phi _{mix} = Y_{air}\phi _{air} + Y_{H_2O}\phi _{H_2O} \end{aligned}$$

2 $$\begin{aligned}{} & {} Y_{air} +Y_{H_2O} = 1, \end{aligned}$$

where $Y_{air}$, $Y_{H_2O}$ are the species mass fraction, and $\phi _{air}$, $\phi _{H_2O}$ are the diffusion flux of dry air and water vapor, respectively.

With the Eulerian description, the continuity equations of dry air and water vapor are solved separately to obtain the transport characteristics of the ambient airflow field:

3 $$\begin{aligned}{} & {} \frac{\partial }{\partial t}\left( \rho _{mix}Y_{air}\right) + \nabla \cdot \left( \rho _{mix}Y_{air}\overrightarrow{U}_{mix}\right) = 0 \end{aligned}$$

4 $$\begin{aligned}{} & {} \frac{\partial }{\partial t} \big [\rho _{mix}Y_{H_2O} + \nabla \cdot \left( \rho _{mix}Y_{mix}\overrightarrow{U}_{mix}\right) + \nabla \cdot J_{H_2O} \big ] = S_{H_2O} \end{aligned}$$

5 $$\begin{aligned}{} & {} J_{H_2O} = - \rho _{mix}D_{k}\left( \nabla Y_{H_2O}\right) , \end{aligned}$$

where $\overrightarrow{U}_{mix}$ is the mass-averaged mixture velocity, $S_{H_2O}$ is the mass source of water vapor due to saliva droplets evaporation, $J_{H_2O}$ and $D_{k}$ are the diffusion flux and the kinematic diffusivity of water vapor in the air mixture, respectively.

The ideal mixture between dry air and water vapor is assumed to share the same local velocity, pressure, and temperature. Hence the conservation equations of momentum and energy are combined into the mixture form:

6 $$\begin{aligned}{} & {} \frac{\partial }{\partial t} \left( \rho _{mix}\overrightarrow{U}_{mix}\right) + \nabla \cdot \left( \rho _{mix}\overrightarrow{U}_{mix}\overrightarrow{U}_{mix}\right) = \overrightarrow{F_{md}} - \nabla P + \nabla \cdot \tau + S_{Buoy} \end{aligned}$$

7 $$\begin{aligned}{} & {} \frac{\partial }{\partial t} \left( \rho _{mix}E\right) + \nabla \cdot \left[ \overrightarrow{U}_{mix}\left( \rho E + P\right) \right] = \nabla \cdot \left[ k\nabla T - h_{H_2O} J_{H_2O}+ \left( \tau \cdot \overrightarrow{U}_{mix}\right) \right] + S_{h}, \end{aligned}$$

where $\overrightarrow{F_{md}}$ is the interfacial force incurred by the droplet particles, $\tau$ is the viscous stress tensor caused by the viscous effect, and $S_{Buoy}$ is the momentum source due to buoyancy. *P*, *E*, *k*, *T*, and $\mu _{mix}$ are the pressure, energy, thermal conductivity, temperature, and viscosity of the mixture air, respectively. $S_{h}$ is the volumetric heat source. The first three terms on the right-hand side represent heat conduction, species diffusion, and viscous dissipation.

#### Movement of discrete phase

In order to model the particle-laden flow, DPM within ANSYS Fluent is induced to capture the trajectory of each saliva droplet particle produced by the coughing process at each time interval. DPM treats the discrete Lagrangian phase as a continuum structure so that the transient properties of particles (velocity, mass, and position) are computed by integrating the force balance. In light of the sizeable droplet-to-air density, the interfacial forces due to the density ratio are negligibly small. Following Newton’s second law, the equation of motion for particles mainly considers the effect of gravity, stokes-Cunningham drag force, Brownian motion-induced force, and buoyancy, i.e.,

8 $$\begin{aligned}{} & {} \frac{d}{dt}\left( m_{dro}\overrightarrow{U_{d}}\right) = \overrightarrow{F_{G}} + \overrightarrow{F_{D}} + \overrightarrow{F_{B}} + \overrightarrow{F_{Buoy}} \end{aligned}$$

9 $$\begin{aligned}{} & {} \overrightarrow{F_{G}} = m_{dro}g\left( 1 - \frac{\rho _{dro}}{\rho _{mix}}\right) \end{aligned}$$

10 $$\begin{aligned}{} & {} \overrightarrow{F_{D}} = \frac{1}{8C_{c}}\phi \rho _{mix} d_{dro}^{2}C_{D}\left( \overrightarrow{U} - \overrightarrow{U_{dro}}\right) \left| \overrightarrow{U} - \overrightarrow{U_{dro}}\right| , \end{aligned}$$

where $m_{dro}$, $\overrightarrow{U_{dro}}$ and $d_{dro}$ are droplet mass, velocity, density, and diameter, respectively. $C_{c}$ is Cunningham correction factor^58^. The value of the drag coefficient ($C_{D}$) depends on the droplet’s Reynolds number ($R_e$)^59^:

11 $$\begin{aligned} C_{D} = a_{1} + \frac{a_{2}}{R_e} + \frac{a_{3}}{R_e^{2}}, \end{aligned}$$

where $a_{1}$, $a_{2}$ and $a_{3}$ are constants which be decided by the range of $R_e$.

#### Evaporation of discrete phase

It is assumed that droplets remain spherical during evaporation. According to^49^ in Table 3, the conclusion is that the actual nuclei size depends on the droplet composition. Droplet particles are selected as a combination of 98.2% volatile content pure water and 1.8% non-volatile solid compounds (including the COVID-19 virus). Each component is implanted into the droplet model through user-defined commands. According to the measurement of Silva et al.^60^ from infected saliva samples, the average COVID-19 viral load of males and females is about 5.4 $\hbox {log}_{10}$ G.E. (viral genome equivalents)/ml and 6.2 $\hbox {log}_{10}$ G.E./ml, where 1 G.E. equals 6.6 picograms of DNA. The proportion of COVID-19 virus in saliva droplets is presumed to be 6.6e−6 g/ml with the same molecular weight as DNA. According to the experimental data measured by Lieber et al.^35^, although the non-volatile components cause a decrease in the vapor pressure of droplets, the evaporation rates of droplets made up of pure water and saliva are nearly identical in a duration of 58 s. Hence, only the water component evaporates until the droplet particle reduces to its nuclei during condensation and evaporation. The mass transfer between ambient vapor and pure water in droplets solves the rate of evaporation:

12 $$\begin{aligned} \frac{dm_{dro}}{dt} = k_{c}A_{dro}\rho _{dro}ln\left( \frac{1 - Y_{H_{2}O,s}}{1 - Y_{H_{2}O,mix}}\right) , \end{aligned}$$

where $k_{c}$ is the mass transfer coefficient, $A_{dro}$ is the surface area of droplet particles. $Y_{dro, s}$ and $Y_{dro, mix}$ are the equilibrium water vapor mass fraction at the droplet surface and the local water vapor mass fraction in the mixture air.

In light of the minor heat change inside the droplet, the energy balance equation only took into consideration the latent heat of phase change is expressed by:

13 $$\begin{aligned} \frac{d}{dt}\left( m_{dro}c_{dro}T_{dro}\right) = hA_{dro}\left( T_{\infty } - T_{dro}\right) + \frac{dm_{dro}}{dt}h_{lH_{2}O}, \end{aligned}$$

where $c_{dro}$ is the droplet heat capacity, *h* is the convective heat transfer coefficient, $T_{\infty }$ is the temperature of surrounding mixture air, and $h_{lH_{2}O}$ is the latent heat.

#### Schlieren experimental setup

**Figure 15:**
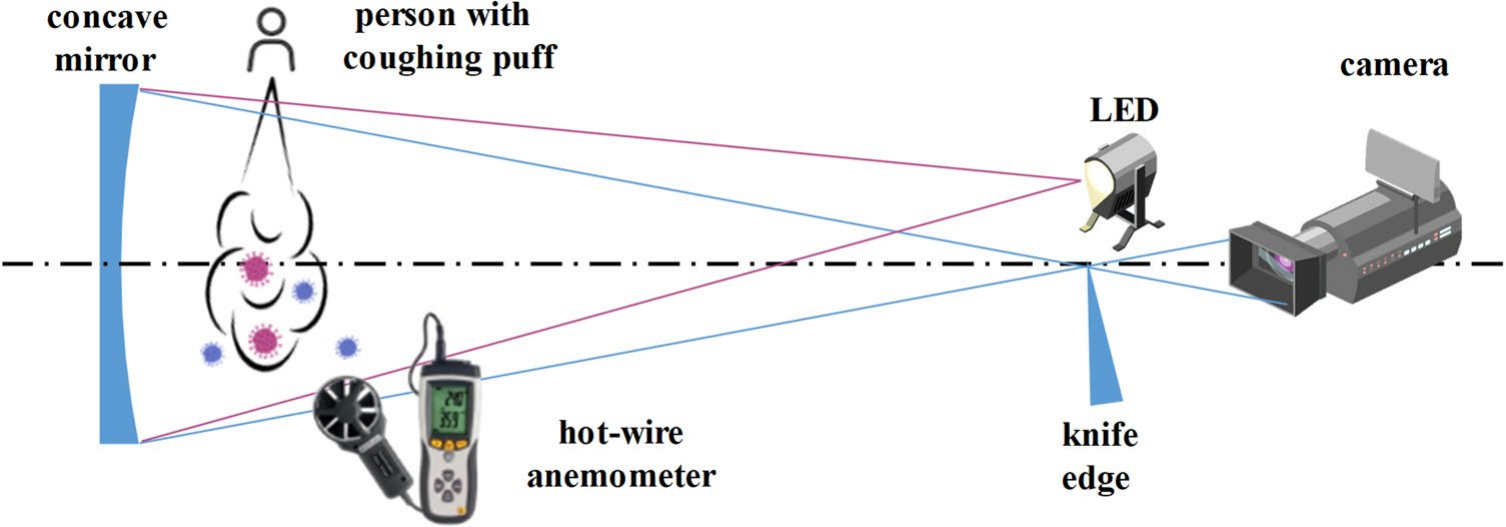
Components of the Schlieren imaging system. The figure was drawn in Adobe Illustrator (version 16.0).

The principle underlying the Schlieren experiment is that the shadowgraph imaging technique based on the refractive indices of light will be different when passing through warm air with various temperatures. As shown in Fig. 15, the light from a LED is arranged in a double pass coincident setup consisting of a concave mirror with 203 mm diameter and 750 m focal length and a camera with frame rates ranging from 30 to 240 fps. The test subject is made to cough from a distance of 0.1 m from the center of the concave mirror. The motion of the cough wavefront and the turbulent features of the puff are captured by the camera. Meanwhile, horizontal airspeed at 0.2 m far from the test subject is measured using a hot-wire anemometer, which has an accuracy of $\pm \, 5\%$ of the measured value and a measurement range of 0.01–30 m/s.

**Figure 16:**
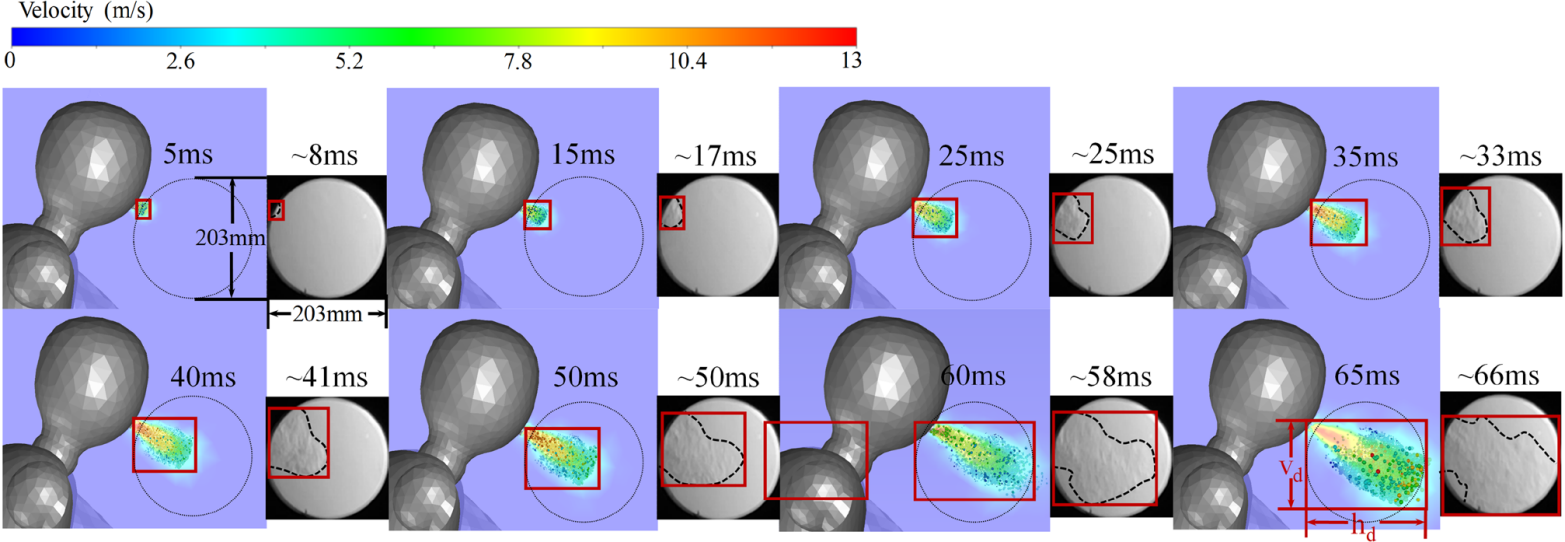
Typical cough without from CFD and Schlieren experiment. The figure was processed by Ansys Fluent (version 2019 R2) and drawn in Adobe Illustrator (version 16.0).

**Figure 17:**
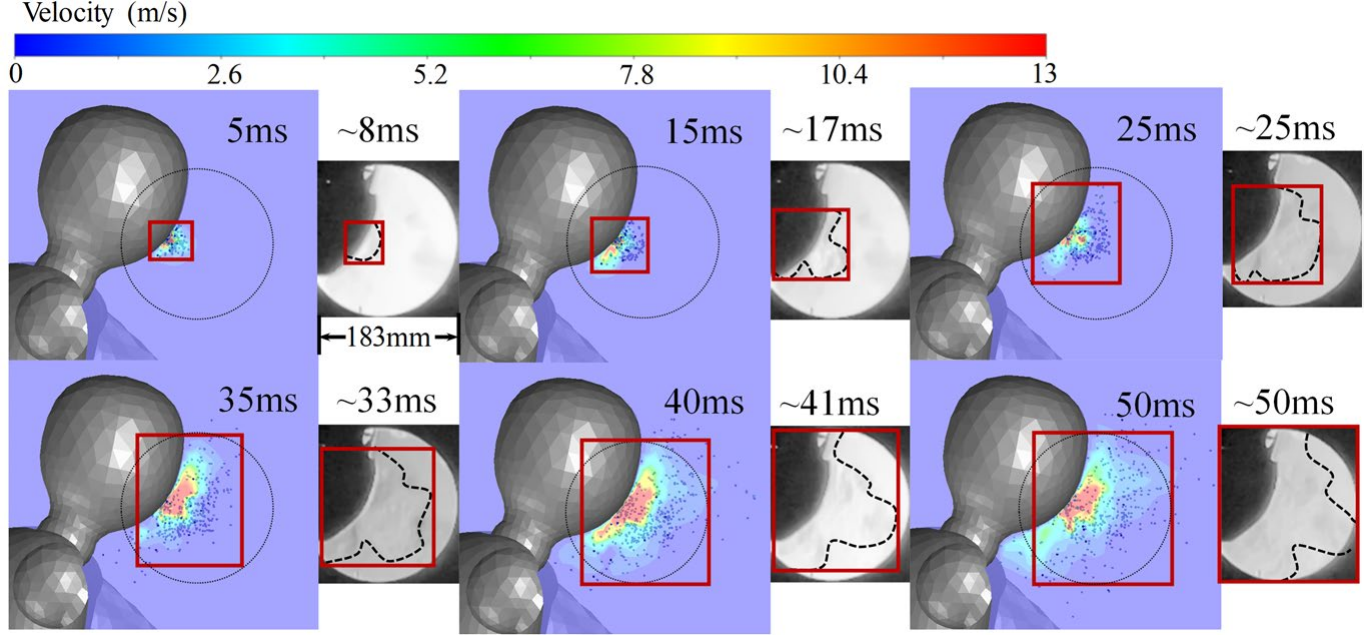
Typical cough with single-layer gauze mask from CFD and Schlieren experiment. The figure was processed by Ansys Fluent (version 2019 R2) and drawn in Adobe Illustrator (version 16.0).

**Figure 18:**
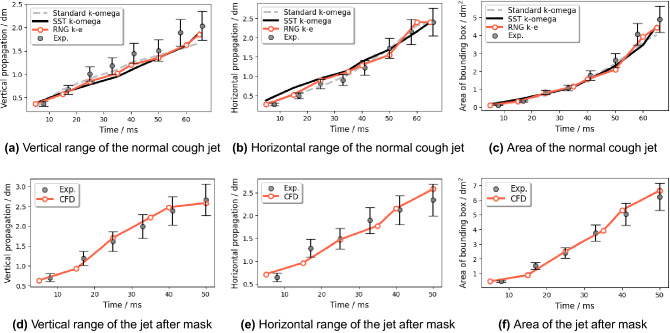
The comparison of cough propagation range between the CFD result of Cases 1 (normal cough)/Case 2 (cough with gauze mask) and Schlieren experimental results.

## Simulation and results

### Experimental validation

In order to verify the evaporation model, the prior Lagrangian evaporation model of a single water droplet interacting with air in ANSYS Fluent is simulated to compare with two series of experimental data. Typical parameters in the first experimental^57^ setup are: $\hbox {R.H.} = 35\%$, air temperature = 297 K, free-stream velocity = 0.203 m/s, and droplet initial diameter = 1.05 mm. The same conditions are repeated in the CFD simulation. During a period of 800 s, the CFD predicted change of the droplet diameter is recorded and plotted with experimental results in Fig. 12. For the second experiment^57^, the ambient air temperature is 298 K, the airflow velocity is less than 0.1 m/s, the initial diameter of the droplet is 1337 $\upmu \,\hbox {m}$, and the R.H. is $96\%$ (Fig. 13) and $84\%$ (Fig. 14), respectively. Both experiments and simulations in Fig. 13 are aborted when droplets are totally evaporated, but the droplets roughly maintain their initial masses all the time under the $\hbox {R.H.} = 96\%$ circumstance. It is observed that the mass of both the water droplets in the circumstances with high R.H. will roughly maintain their masses when the phase equilibrium is reached at the droplet surface. A satisfactory agreement is obtained between the numerical results of this study and the experimental data reported in the literature. The figure also reveals that the total mass of airborne droplets is sensitive to ambient conditions. The curvature of the black line is getting increased, which declares the larger droplets with a larger specific surface area lead to a slower reduction in diameter due to evaporation.

The posterior validation analysis is based on the images acquired from the Schlieren experiment, whose facilities are shown in Fig. 15. At the indoor temperature and R.H. condition (20 $\circ \hbox {C}$ and $48\%$), the maximum visible propagation distance from the boundary of the grayscale Schlieren images to the coughing wavefront and the maximum visible 2-dimensional area of puff in the experiment both have a small extension than that of numerical simulations, while the airspeed is smaller than the CFD result. The comparisons in Figs. 16 and 17 show an exceptionally good precision of the above-mentioned CFD model for both qualitative analyses of the transportation of coughing droplets.

The quantitative comparison in the propagation range of the experimental cough jet with or without a mask in vertical, horizontal, and areal dimensions between the results of the Schieren experiment and CFD is shown in Fig. 18. Limited by the size of the concave mirror, the Schlierence experiment can only validate the first 65 ms during the normal coughing process and 50 ms with a single-layer gauze mask. As shown in Fig. 18a–c), the results simulated from the RNG k-e turbulence model show a better match and a higher accuracy than the other two CFD results from Standard k-omega and SST k-omega. The results of the normal cough show that the CFD result with the RNG k-e turbulence model is lower than the experimental one on the vertical range. In contrast, the horizontal range is larger, of which the combined effect is the areal result from CFD comparatively fitting well with the experimental one. For the masked cough jet (Fig. 18d–f), the CFD results are consistent with the experimental ones. Overall, the CFD results based on the RNG k-e turbulence model are reliable.

### Spatial and temporal effect on an indoor cough process

**Figure 19:**
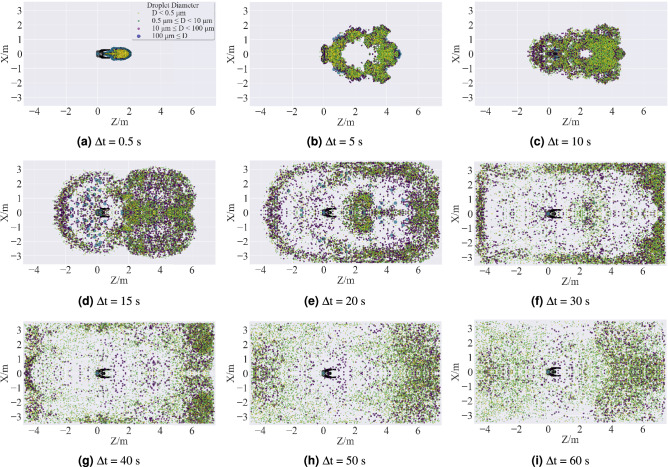
Top view of cumulative time distribution of droplet clouds in Case 1. The distributions of droplets were predicted by using the ANSYS Fluent (version 2019 R2). The figures were drawn by Matplotlib (version 3.5.0).

**Figure 20:**
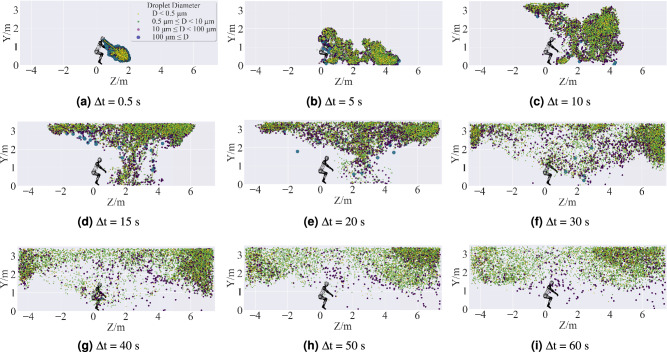
Side view of cumulative time distribution of droplet clouds in Case 1. The distributions of droplets were predicted by using the ANSYS Fluent (version 2019 R2). The figures were drawn by Matplotlib (version 3.5.0).

**Figure 21:**
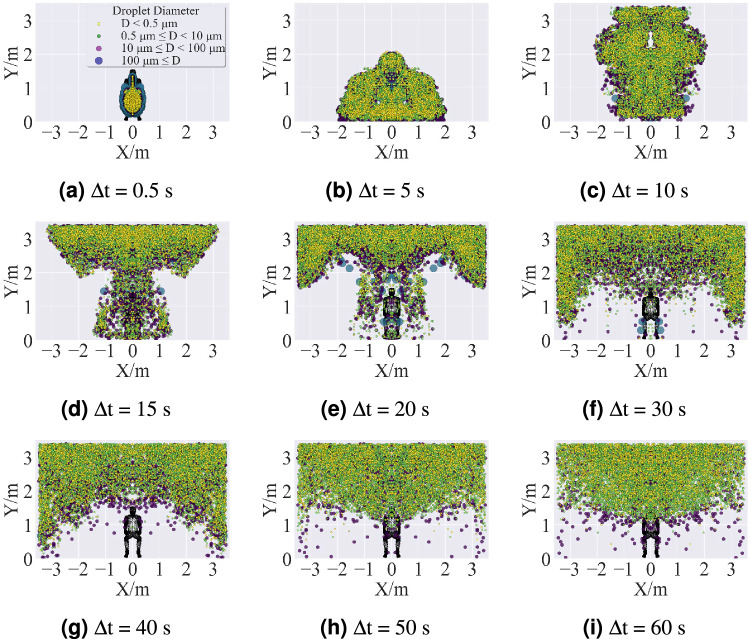
Front view of cumulative time distribution of droplet clouds in Case 1. The distributions of droplets were predicted by using the ANSYS Fluent (version 2019 R2) and drawn by Matplotlib (version 3.5.0).

Figures 19, 20 and 21 are, in order, top view, side view, and front view of the droplet distribution in the classroom to analyze the spatial dispersion of the droplet cloud for a normal cough in quasi-quiescent air. For Case 1, the baseline pattern of a regular cough is obtained at $\Delta \hbox {t} = 0.5$ s, which is different from the presupposed conical spray. At this moment, all the virus-burden saliva droplets have been released from the mouth, and a part of them has already begun to evaporate. Those droplets just sprayed out are traveling alone straight line because they obtained momentum and energy from the cough jet source. In contrast, the early residual droplets lost their momentum and kinetic energy quickly and began to dissipate peripherally under the effect of drag force. After 5s from the onset of the cough, the larger droplets (D $\ge$ 100 $\upmu \,\hbox {m}$) deposited on the ground under the action of gravity, and the smaller droplets further dissipated due to turbulence. During the time from 10 to 50 s, aerosol formed and ascended to the ceiling with the thermal plume from the human body, then scattered and spread to the surrounding. Eventually, the aerosol consisting of non-volatile nuclei (D < 0.5 $\upmu \,\hbox {m}$) and smaller droplets (0.5 $\upmu \,\hbox {m}$ $\le$ $\hbox {D} < 10$ $\upmu \,\hbox {m}$) remains suspended in the whole half-top air long enough, with a few larger droplets (10 $\upmu \hbox {m} \le$ D < 100 $\upmu \hbox {m}$) hindered settling. During the whole process, the values of Stokes number of saliva stay much lower than 1, which means the inertial effect of a particle is weak and, consequently, the inertia settlement is not prominent.

**Figure 22:**
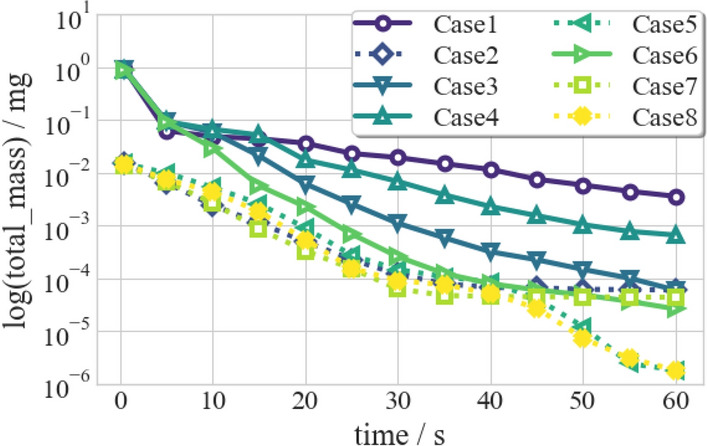
Total mass of airborne droplets varies with time.

**Figure 23:**
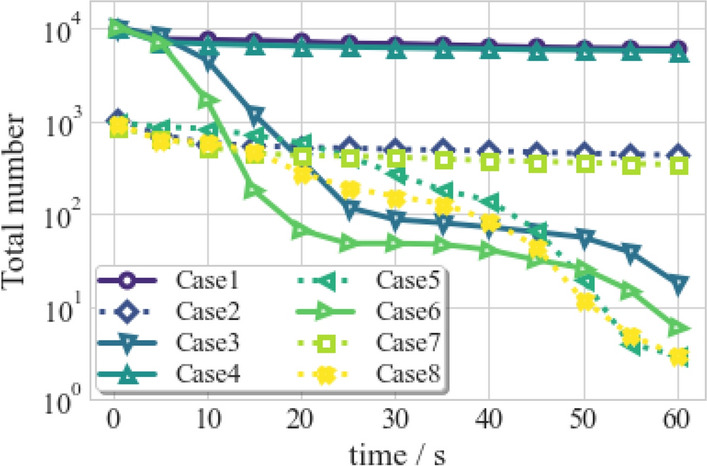
Total number of droplets varies with time.

The total mass ($m_{dro,t}$) of airborne droplets as a quantitative parameter of the transmission and dissipation of the droplets in the air is defined by:

14 $$\begin{aligned} {m_{dro,t} = \sum _{i=1}^{n} m_{dro,i} = \sum _{i=1}^{n} (m_{v,i} + m_{nonv,i})}, \end{aligned}$$

where $m_{dro, i}$ is the mass of an individual particle, $m_{v, i}$ and $m_{nonv, i}$ are the mass of the water component, and the non-volatile species in a particle, respectively. $m_{v, i}$ diminishes gradually over time due to the mass transfer effect described in Eq. (12), while $m_{nonv, i}$ is invariant with time and is determined by the $1.8\%$ initial mass of an individual droplet. Figure 22 shows the change in $m_{dro,t}$ of airborne droplets over time for the eight cases. The solid purple line represents Case 1, which typically indicates a downward trend over time. The descent is steep in the first 5 s, then flattened. Two motions of droplets cause this phenomenon: evaporation and settlement. Its rate is strongly affected by ambient thermal conditions and weakly by turbulent mixing for evaporation. Therefore, the evaporation process is decoupled with other mechanisms and continues to reduce the total mass. For settlement, in the early 5 s, gravitational and inertial acceleration balance with the drag force and buoyancy, dominating the rapid fall of larger droplets. Once the droplet settles, its mass is removed from the computational domain. Thereafter, diffusion and inertia settling is pronounced among the droplet aerosol, which results in less removal of total droplet mass.

## Discussions of COVID-19 precautions

### Droplet concentration

**Figure 24:**
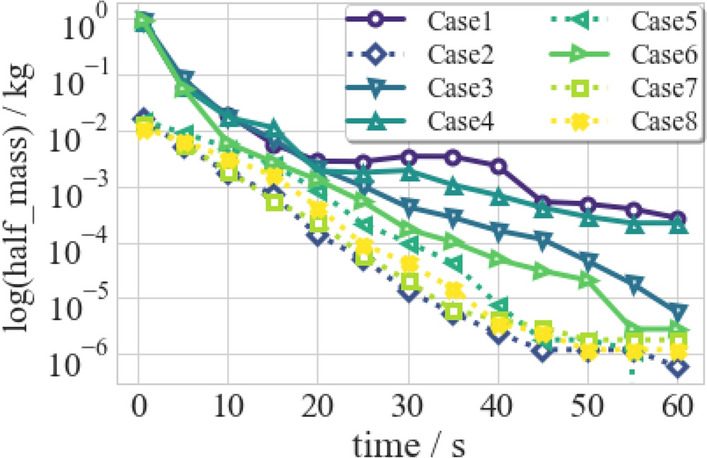
The total mass of droplets in the lower half of the classroom varies with time.

In this study, the precautions adopted in Case 2–Case 8 are applied before the coughing process, so all the cases have different steady initial aerial conditions. The handling of the completely evaporated droplets in this paper is regarded as non-volatile solid nuclei to analyze the distributions of residual saliva. We compare the effect of preventative strategies, including face masks, upward ventilation, and protective screen.

In accordance with Wells–Riley mathematical model for infection probability, aerosol density and distance play an essential role in the infection risk. Their total mass and total number can analyze the density of respiratory droplets. For the total mass, Fig. 22 indicates that Case 5 and Case 8 have the least risk of infection. Many large droplets are deposited in the action of gravity rapidly, and small droplets are accelerated by the suction of ventilation. However, the medium droplets will suspend in the air for a longer time because the gravity, buoyancy, and drag force they suffer from will counteract the suction from ventilation. Face mask (Case 2/5/7/8) with filtration effect can reduce the total mass of the coughing cloud and the number of large droplets in the initial stage. Hence, the medium droplets with the longer suspension time dominant in Case 2/5/7/8, the change of total mass in these four cases mainly caused by vaporization follow almost the same curve and differ from the other four cases before 45 s. After 45 s, the suspending droplets in Case 5 and Case 8 travel to the ceiling and will be expelled from the ventilation outlet, while droplets in Case 2 and Case 7 will continue to suspend. For the total number, the line chart in Fig. 23 indicates Case 3 and Case 5 plunge the droplet number in the early 20 s because the venting system increases the momentum of droplets. All the ventilated cases (Case 3/5/6/8) reduce the number of droplets at the final time in the classroom. The indoor upward ventilation pattern is the most effective strategy and is essential in safeguarding against COVID-19.

In daily life, the lower half of the classroom is the main activity space for human beings. Figure 24 shows the total mass of droplets in the lower half of the classroom ($m_{lh}$). In Case 1, $m_{lh}$ initially decreases, increases slightly, and declines again. The intermediate increase is due to the settlement of the suspending aerosol. After wearing the face mask (Case 2/5/7/8), especially in the early period of cough, the concentration of droplets with COVID-19 is reduced sharply. Moreover, the ventilated cases (Case 3/6) eliminate the intermediate increase. It is indicated that all the precautionary strategies can reduce the risk of direct inhalation of pathogenic droplets in the activity space of the classroom.

### Propagation range of droplets carrying COVID-19

**Figure 25:**
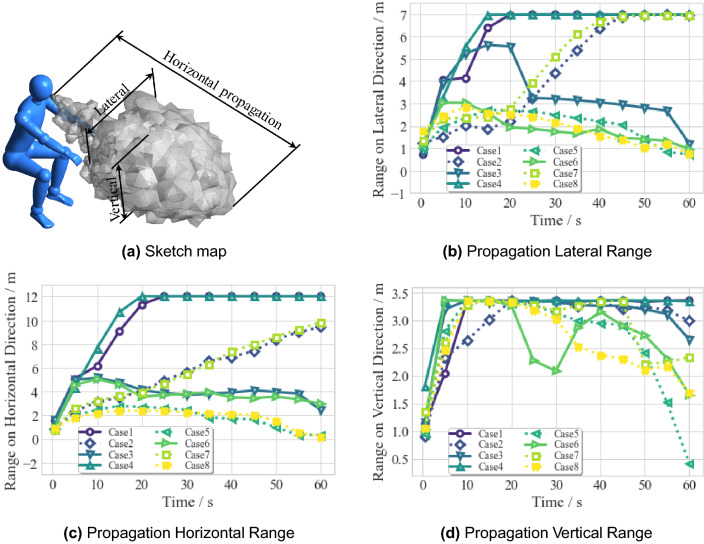
Propagation range of droplet cloud varies with time. (**a**) Drawn in Tecplot 360 (version 2016 R2) and Adobe Illustrator (version 16.0).

Figure 25 shows the propagation range in the three dimensions from the spatial distribution of the discrete droplet cloud under the perspective of the Lagrangian approach. Figure 25B shows the maximum range of lateral penetration varying with time. The range in Case 1/2/4/7 is gradually increasing to cover the whole lateral span of the classroom, while the cases implemented by the upward ventilation (Case 3/5/6/8) have lesser dispersing ranges (as far as 1 m). That verifies one of the conclusions of Lieber^35^: a safe distance cannot be defined for an indoor scenario without sufficient ventilation—droplets will spread the whole room.

**Table 4:** Recommended social distance on cases with upward ventilation.

Case no.	Horizontally (m)	Vertically (m)
3 (ventilation)	6	6
5 (mask and ventilation)	3	3
6 (ventilation and screen)	6	3
8 (mask and ventilation and screen)	3	3

Figure 25C shows the horizontal penetration of the droplet cloud. Again, the droplets in Case 1 and Case 4 finally be projected to the whole horizontal span of the room, whereas both Case 5 and Case 8 can project droplets to the peak of 3m of their range at $\Delta t = 15$ s then decrease, and finally approximately equal to 0 m. The comparison shows that Case 5 and Case 8 have the least infection risk.

Figure 25D shows the magnitude of the vertical penetration. For all cases, the tendency of their ranges is similar (increasing to the maximal height of the classroom—3.35 m) during 0.5–20s. However, the following higher differences are induced by the various preventative strategies. In general, the cases (Case 5/6/7/8) with the coupling effect of two or three strategies have a smaller range of propagation. Case 5 is the optimum at the destination, with a range of less than 0.5 m. Compared with Case 5, Case 8 is suboptimum because its additional protective screen negatively affects the airflow field: the screen blocks the venting air, and a few droplets with low momentum are stuck in the recirculation flow upon the screen.

Since saliva will finally spread to the whole space in a room without ventilation, the recommended social distance makes little sense in such a limited enclosed space. In Table 4, social distancing guidance is shown on the occasion of the four cases with ventilation. To sum up, for well a ventilated room (Case 3), the recommended social distance is 6 m; the protective screen combined with upward ventilation (Case 6) can reduce the horizontal social distance by 3 m; when using a face mask and ventilation simultaneously (Case 5 and Case 8), the social distance is 3 m both in horizontal and vertical.

### Probability density function of droplet size distribution

**Figure 26:**
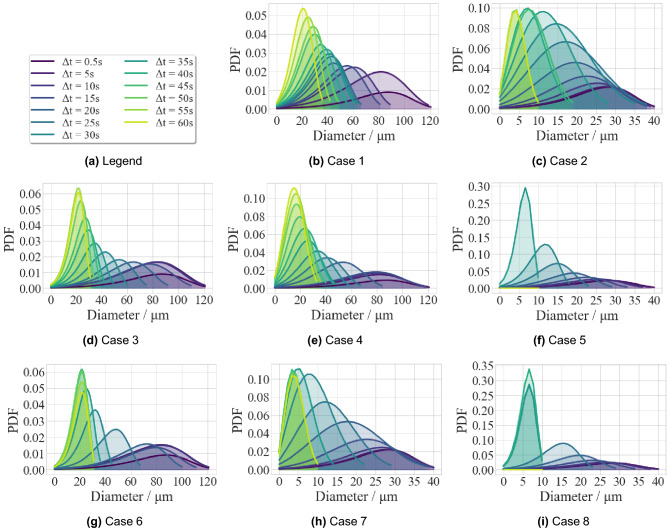
PDF varies with time.

Due to evaporation and settling, the droplet size in the air changes with time when the droplets travel through the air. The droplet size distribution is fitted to the probability density function (PDF) of the Rosin-Rammler approach based on the computational results for the 8 cases, as shown in Fig. 26. For all cases, the interval and mode of the PDF decrease over time. The large droplets (D $\ge$ 100 $\upmu \,\hbox {m}$), in cases without masks (Case 1/3/4/6), fall on the ground within the same time interval (less than 20 s). As aforementioned, although the gravitational settlement is dominant in the early stage, evaporation works all the time and eventually overcomes the settlement, dominating the decrease in droplet size. In the face-mask scenarios (Case 2/5/7/8), the size of droplets decreases, and the gravitational settlement vanishes, because the PDF is mainly affected by the mass transfer due to evaporation at this time (and also ventilation in Cases 5 and 8).

The functions and limitations of upward ventilation and protective screen with and without a mask are yielded below. Without the mask, the ventilation scenario (Case 3) provides an accelerated speed contrary to the gravitational acceleration to help tiny droplets escape from the ceiling, which is proved by the accelerating shrinkage of the PDF distribution during the later period (from the 20 s to 45 s). Utilizing a protective screen alone (Case 4) is effective in preventing the widely diffused droplets with higher initial momentum by rebounding them upward to the ceiling. Under the combined action of a mask, the large droplets are filtrated, and only medium and small droplets are left. Thus the benefit of ventilation (Case 5/6/8) becomes prominent by increasing the expelling of droplets. For the combined cases with mask and screen (Case 7 and Case 8), however, the less range of cough jet causes the futility of screen.

### Effect of ACH on concentration and propagation of droplets

**Figure 27:**
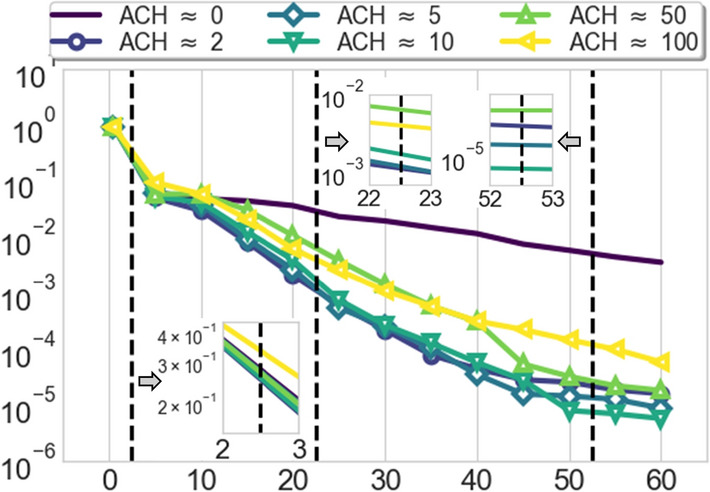
Total mass of airborne droplets under various ACH.

The importance of ACH is that it can strongly affect the concentration and propagation of droplets, which is a significant parameter when designating precautious guidance for closed indoor scenarios. As suggested by previous research, increasing ACH from 4 to 12 ACH impact little on the infection risk^61^, and the stronger air movement (ACH = 40) can prevent the propagation of the coughing aerosol more effectively^10^. Conditioned air at various specified operation requirements are different^62^, for instance, 6–20 ACH for classrooms, 10–50 ACH for precision manufacturing, etc. The present research investigates the effect of ACH on the concentration and propagation of droplets by reducing the air velocity at the inlet in Case 3 (ACH $\approx$ 100) to 0.002 m/s (ACH $\approx$ 2), 0.005 m/s (ACH $\approx$ 5), 0.01 m/s (ACH $\approx$ 10), and 0.05m/s (ACH $\approx$ 50), respectively. As shown in Fig. 27, the decrease of ACH leads to the reduction of the total mass of droplets in the final step of the simulation. Compared to the case of ACH $\approx$ 0 (Case 1), the remarkable reductions in concentration caused by ventilation (ACH $\ge$ 0) appear after 10s. The total mass of coughing droplets under ventilation is characterized in different periods by three main physical mechanisms: settlement, evaporation, and escape. Three typical time points of 2.5 s, 22.5 s, and 52.5 s represent the earlier stage (gravitational settlement domain, combined with evaporation), medium stage (evaporation and escape domain, combined with the weaker gravitational settlement), and later stage (escape domain, combined with the weaker evaporation, diffusion, and inertial settlement), respectively, intercept the numerical duration by dashed lines. At 2.5 s, lines 0–50 coincide with each other, but the total mass in the case of 100 is the largest. Since the upward ventilation with the highest velocity weakens the gravitational settlement of the droplets with larger diameters, the remained larger droplets cause a higher total mass in the case of 10. At 22.5 s and 52.5 s, the total mass of droplets in the cases with the lower ACH (ACH $\approx$ 2, 5, and 10) is the least. Moreover, the case of 2 and case of 5 slightly work better than the case of 10 at 22.5 s, while the case of 10 has the best effect at 52.5 s. The higher ACH will cause a negative effect at the earlier and medium stages, but the larger ACH has a greater efficiency in exhausting the droplets when the gravitational settlement vanishes. To sum up, 10 is the recommended value of ACH with the best effect on the reduction of the concentration of droplets in the long term.

**Figure 28:**
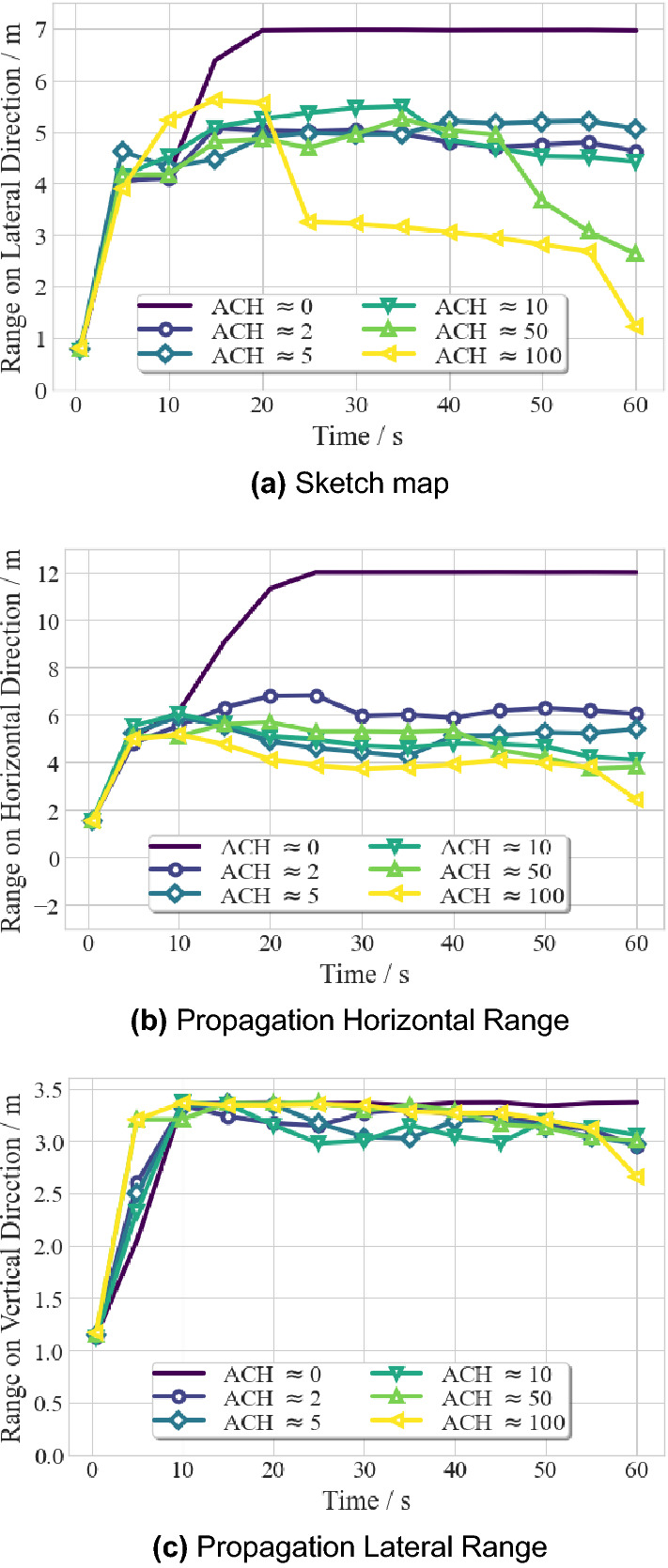
Propagation range of droplet cloud varies with time.

As shown in Fig. 28, the horizontal and lateral ranges (Fig. 28a,b) of the cough jet in the cases of ACH $\approx$ 2, 5, 10, and 50 are broadened than ACH $\approx$ 100. The larger ACH can diminish the propagation range, but when the ACH $\le$ 10, the values and tendencies of the propagation ranges are similar. For the vertical ranges (Fig. 28c), the curve of 100 gradually declines after the 30 s and becomes lower than others at 60 s. Since the ACH of 100 infuses droplets with a larger vertical velocity, which limits the horizontal and lateral diffusion and accelerates the vertical exhausting of droplets. Overall, a larger ACH is recommended for upward ventilation to reduce the indoor propagation range of cough droplets.

## Conclusions

A multi-component Eulerian–Lagrangian CFD particle-tracking model with user-defined functions is proposed in this study to quantitatively describe the evaporation and dispersion of COVID-19 laden droplets in an enclosed room. The model features mass, momentum, and heat transfer between discrete cough droplets and continuous background air. The user-defined functions reinforce the transient feature of coughing progress. The model predictions are in good agreement with the experimental data. Critical effects of case-specific precautions (e.g., face mask, upward ventilation, protective screen, and combinations) are focused on and evaluated. The conclusions arising from this study are as follows:

1. In terms of a normal cough, the evolution of the cough droplets is mainly affected by two mechanisms—evaporation and settlement. Gravitational settlement predominates in the early stages, especially for larger water droplets. In contrast, diffusion coupled with inertial settling predominates in the middle and later stages, more pronounced for smaller water droplets. Evaporation happens on all-size droplets throughout the duration, so the larger droplets decrease in size, and airborne droplets become small enough to suspend in the higher airways.
2. For cases where a single precaution is implemented, upward ventilation is the most effective measure, followed by the face mask and then the protective screen. Upward ventilation forces the droplet cloud to follow the venting air and reduces infector risk overall. A face mask can control the diffusion of the cough jet from the source and filter the droplets with a larger size. A protective screen can prevent gravitational settlement and direct the cough cloud away from human activity space to the ceiling.
3. Case 5 (mask + ventilation) and Case 8 (mask + ventilation + screen) work equally well, so both are relatively optimal solutions. In these two cases, the number density of airborne droplets and the mass concentration of inhalable aerosol can be significantly decreased (by $\sim \,99.95\%$ in number and $\sim \,99.95\%$ in mass, compared to Case 1), resulting in a shorter propagation distance and a lower infection risk in the human activity space. It is also found that the protective screen becomes dispensable when used with a face mask.
  with the best effect on the reduction of the concentration of droplets
4. The indoor upward ventilation pattern plays the most significant role in safeguarding against COVID-19. The ventilation with an ACH of 10 lower the concentration of droplets to most effectively, and a larger ACH is recommended to reduce the indoor propagation range of cough droplets. For an individual, wearing face masks appears as the most convenient and efficient solution to limit the spread of COVID-19. Indeed, many people are not correctly wearing their masks or wearing masks with weak filter capability. In these cases, the protective screen could be the secondary protection to lower the infector risk of COVID-19.
5. Face mask and protective screen reduce the front jet flow, which causes less harm in face-to-face circumstances; upward ventilation is used in heating mode to avoid complete mixing. The recommended social distance for a classroom scenario with the combination of mask and ventilation is 3 m, horizontally and vertically.

Further research is needed to attain more effective ventilation and screen strategies that can take advantage of new technology like A.I., with detailed scenarios taken into account, such as tables coating adsorption materials, windows to convect air naturally, and incorrectly masked humans, for a better response to future epidemic diseases.

## Data Availability

The datasets and materials used and/or analyzed during the current study are available from the corresponding author on reasonable request.
